# Characteristics of Malignant Pleural Effusion Resident CD8^+^ T Cells from a Heterogeneous Collection of Tumors

**DOI:** 10.3390/ijms21176178

**Published:** 2020-08-27

**Authors:** Rajeev Dhupar, Olugbenga T. Okusanya, Seth H. Eisenberg, Sara E. Monaco, Ayana T. Ruffin, Dongyan Liu, James D. Luketich, Udai S. Kammula, Tullia C. Bruno, Michael T. Lotze, Adam C. Soloff

**Affiliations:** 1 Surgical Services Division, VA Pittsburgh Healthcare System, Pittsburgh, PA 15240, USA; 2 Department of Cardiothoracic Surgery, University of Pittsburgh School of Medicine, Pittsburgh, PA 15213, USA; okusanyaot@upmc.edu (O.T.O.); she14@pitt.edu (S.H.E.); luketichjd@upmc.edu (J.D.L.); acs202@pitt.edu (A.C.S.); 3 Department of Pathology, University of Pittsburgh School of Medicine, Pittsburgh, PA 15213, USA; monacose@gmail.com; 4 Department of Immunology, University of Pittsburgh School of Medicine, Pittsburgh, PA 15213, USA; atr30@pitt.edu (A.T.R.); dongyan@pitt.edu (D.L.); tbruno@pitt.edu (T.C.B.); lotzmt@upmc.edu (M.T.L.); 5 Department of Surgery, Division of Surgical Oncology, University of Pittsburgh School of Medicine, Pittsburgh, PA 15213, USA; kammulaus@upmc.edu; 6 Department of Bioengineering, University of Pittsburgh School of Medicine, Pittsburgh, PA 15213, USA

**Keywords:** malignant pleural effusion, metastatic cancer, T cell response, tumor immunosuppression

## Abstract

While T cell-based cancer immunotherapies have shown great promise, there remains a need to understand how individual metastatic tumor environments impart local T cell dysfunction. At advanced stages, cancers that metastasize to the pleural space can result in a malignant pleural effusion (MPE) that harbors abundant tumor and immune cells, often exceeding 10^8^ leukocytes per liter. Unlike other metastatic sites, MPEs are readily and repeatedly accessible via indwelling catheters, providing an opportunity to study the interface between tumor dynamics and immunity. In the current study, we examined CD8^+^ T cells within MPEs collected from patients with heterogeneous primary tumors and at various stages in treatment to determine (1) if these cells possess anti-tumor activity following removal from the MPE, (2) factors in the MPE that may contribute to their dysfunction, and (3) the phenotypic changes in T cell populations that occur following ex vivo expansion. Co-cultures of CD8^+^ T cells with autologous CD45^―^ tumor containing cells demonstrated cytotoxicity (*p* = 0.030) and IFNγ production (*p* = 0.003) that inversely correlated with percent of myeloid derived suppressor cells, lactate, and lactate dehydrogenase (LDH) within the MPE. Ex vivo expansion of CD8^+^ T cells resulted in progressive differentiation marked by distinct populations expressing decreased CD45RA, CCR7, CD127, and increased inhibitory receptors. These findings suggest that MPEs may be a source of tumor-reactive T cells and that the cellular and acellular components suppress optimal function.

## 1. Introduction

Facilitating endogenous anti-tumor activity in tumor-specific T cells is the cornerstone of multiple immunotherapy strategies to combat cancer. Checkpoint inhibitor blockade and adoptive cell transfer (ACT) therapies rely on the ability of immune cells to recognize tumor-associated antigens, including neoepitopes, and potentiate T cells to overcome tumor-mediated immunosuppression. However, we do not have a clear understanding of which suppressive factors in the metastatic tumor environment are most critical. Studies of the microenvironment within solid tumors have implicated hypoxia [1,2], nutrient availability [3,4,5], suppressive cytokines [6,7], other molecules (e.g., lactate dehydrogenase (LDH) or lactate) [8,9], and immunosuppressive cell subsets such as regulatory T cells (T_regs_), tumor-associated macrophages (TAMs), and myeloid derived suppressor cells (MDSCs) [10,11,12,13]. Less is known about the impact of these factors within liquid tumor environments such as MPE and malignant ascites.

Malignant pleural effusions (MPE) occur when tumor cells have metastasized to the pleura, resulting in the unopposed collection of fluid in the thoracic cavity, and are a common occurrence in many end stage epithelial cancers. While lung and breast cancer patients represent 70–80% of treated cases, MPEs occur in patients with pancreatic, ovarian, and gastroesophageal cancers, among others. MPEs not only contain cancer cells but can also have a large number of immune cells (>10^8^/L), represented by diverse lymphoid and myeloid subsets. In malignant pleural mesothelioma, a primary tumor of the pleura, tumor samples have an immune signature of elevated T_regs_ and MDSCs that contribute to the anergy of tumor-infiltrating lymphocytes (TILs) and correlate with survival [14]. Notably, the functional capacity of the immune cells in mesothelioma has been demonstrated [15,16,17]. Nevertheless, at about 2500 cases per year in the United States, mesothelioma represents a very small burden of pleural disease when compared with over 150,000 annual MPEs in patients with advanced epithelial cancers.

The number of T cells in proximity to pleural tumor is generally quite large in MPEs, and the study of their potential functionality or conversely, of the cellular and soluble factors in the effusion that contribute to T cell quiescence, has been evolving [15,18,19,20,21,22,23,24]. Even with T cell directed immunotherapy, there are many hurdles the MPE environment might present for successful antitumor activity. Understanding the underlying factors that impact T cell function in an MPE are important to improving current immunotherapies. Therefore, we undertook the present study to examine the reactivity and functional capacity of CD8^+^ T cells isolated from a heterogeneous collection of MPEs of epithelial origin in response to the cellular component of MPEs containing malignant tumor cells. We demonstrate that MPE-resident CD8^+^ T cells possess cytolytic activity and IFNγ production in response to autologous non-hematopoietic MPE cells ex-vivo after 24 h. We identified prominent immunosuppressive MDSC populations and soluble lactate and LDH as potential contributing factors to T cell dysfunction. These findings have implications for understanding the potential efficacy of T cell-targeted therapies for patients with MPEs and provides direction for future exploration of this readily accessible tumor compartment.

## 2. Results

### 2.1. The Immune Composition of MPEs is Heterogeneous and Unique from Peripheral Blood

The cohort presented consists of a variety of primary cancer types, patient ages, and prior treatments, with lung cancers being the most frequent (Table 1). MPEs were of volumes between 300–1000 mm and contained between 8 × 10^7^ and 2 × 10^10^ total cells with an average of 2.33 × 10^9^ ± 1.65 × 10^9^ cells per effusion.

**Figure 1:**
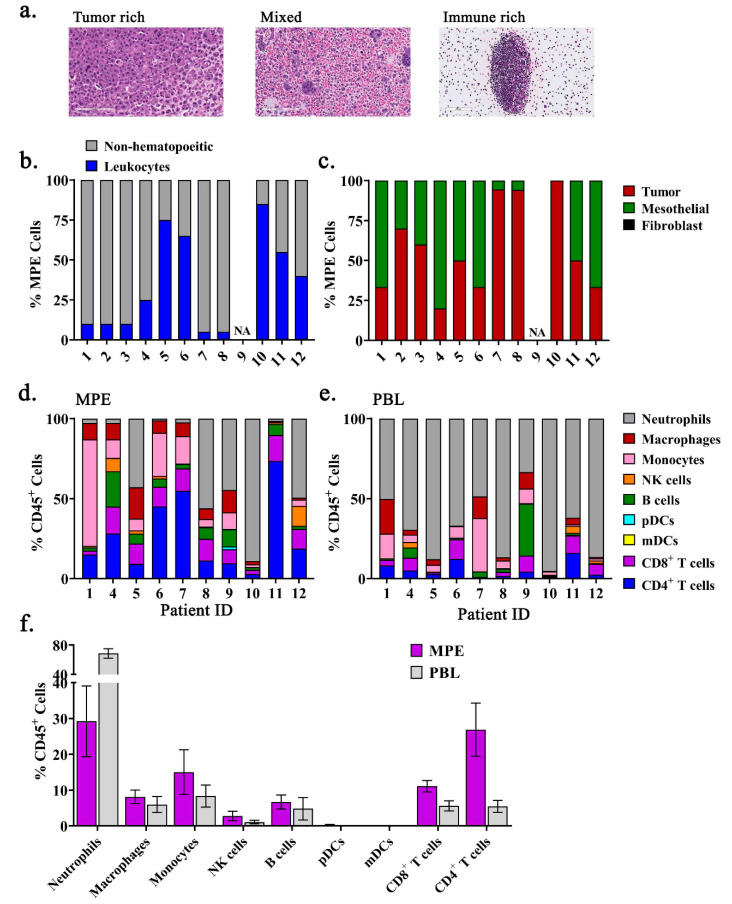
Immunophenotyping reveals heterogeneous composition of MPEs. (**a**) Representative images depicting MPEs rich in tumor cells, immune cells, or a mix of both within cytopathologic specimens. (**b**) Proportions of non-hematopoietic cells and leukocytes in MPEs, and (**c**) representation of tumor cells, mesothelial cells, and fibroblasts (absent) in the non-hematopoietic cell fraction of MPEs. Proportions of neutrophils (grey), macrophages (red), monocytes (pink), NK cells (orange), B cells (green), pDCs (light blue), mDCs (yellow), CD8^+^ T cells (purple), and CD4^+^ T cells (dark blue) from live, single, CD45^+^ leukocytes from (**d**) MPEs or (**e**) peripheral blood leukocytes (PBL) per patient were determined by multiparametric flow cytometry. (**f**) Population means ± SEM of immune cell subsets within MPEs and PBL.

Cytopathologic examination of malignant pleural effusions (MPEs) revealed broad variation in percentage of immune and non-hematopoietic cells, including tumor cells, mesothelial cells, and fibroblasts, with individual MPEs spanning a range from tumor rich to predominantly leukocytes (Figure 1a–c). To examine the immune composition of MPEs, we performed multiparametric flow cytometry to detect neutrophils (SSC^High^CD15^+^), macrophages (SSC^Low^HLD-DR^+^CD11b^+^CD14^−^), monocytes (CD14^+^), natural killer (NK) cells (CD3^−^CD56^+^), B cells (CD3^−^CD19^+^), plasmacytoid dendritic cells (pDCs) (Lineage^―^HLD-DR^+^CD123^+^), myeloid dendritic cells (mDCs) (Lineage^―^HLA-DR^+^CD11c^+^), CD8^+^ T cells (CD3^+^CD8^+^), and CD4^+^ T cells (CD3^+^CD4^+^) from the live, CD45^+^ leukocyte population. Specimen availability precluded analysis of samples 2 and 3, a bilateral MPE obtained from a pancreatic cancer patient, resulting in data on 10 of 12 samples. The composition varied substantially, with individual patient’s possessing immunophenotypes dominated by monocytes, CD4^+^ T cells, or neutrophils (Figure 1d). Notably, there was heterogeneity in the immune composition of MPEs that was not observed within the peripheral blood which consisted predominantly of neutrophils (Figure 1e). The percentage of monocytes and B cells from the peripheral blood positively correlated with those found within the MPE, although both monocytes and B cells were found at lower frequencies in the circulation (Table 2). These findings indicate that the leukocyte composition within MPEs is highly variable and does not reflect the proportion of immune subsets present within the circulation.

### 2.2. A Subset of MPE-Resident CD8^+^ T Cells Possess Tumor-Reactive Functionality Ex Vivo

**Figure 2:**
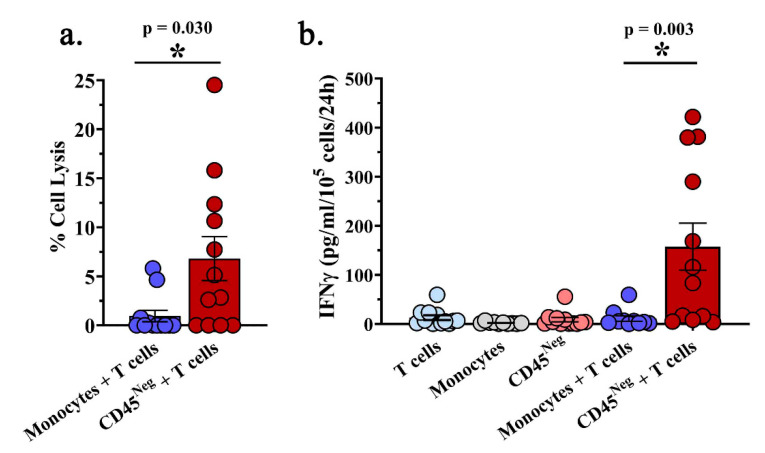
CD8^+^ T cells from MPEs possess functional activity following short-term culture in IL-2. CD8^+^ T cells isolated from MPEs were rested for 24 h in the presence of IL-2, then 10^5^ cells co-cultured at a 1:1 ratio with autologous peripheral blood monocytes or tumor-containing CD45^―^ non-hematopoietic MPE cells for 24 h. (**a**) The percent target cell lysis as determined by lactate dehydrogenase (LDH) cytotoxicity assay is significantly increased following T cell co-culture with non-hematopoietic MPE cells compared to autologous monocyte controls. (**b**) T cells increase production of IFNγ as determined by ELISA in response to autologous non-hematopoietic cells, but not monocytes. IFNγ production from T cells, monocytes, and CD45^―^ non-hematopoietic MPE cells without T cell co-culture was minimal. *p* values of ≤0.05 by Mann–Whitney test were considered significant. Graphs depict mean values with SEM and individual patient determinants.

Next, we examined if MPE-resident CD8^+^ T cells possess antitumor reactivity when removed from the pleural fluid. CD8^+^ T cells were utilized instead of bulk CD3^+^ T cells to circumvent potential immunosuppression by CD4^+^ T_regs_ which were present at high levels (≤ 10% of total T cells and ≤15% of CD4^+^ T cells). MPE-resident CD8^+^ T cells were bead-isolated, rested for 24 h in complete media supplemented with 6000 IU/mL IL-2, and subsequently co-cultured for 24 h at a 1:1 ratio with either autologous tumor containing non-hematopoietic cells from the MPE or autologous peripheral blood monocytes. The percentage of tumor cells in the CD45^―^ fraction ranged from 20–100% (Table S1). Bead enrichment yielded mostly CD8^+^ T cells with minor populations of CD4^+^ T cells and NK cells (Figure S1). This resulted in a significant increase in cell lysis and IFNγ production in the tumor containing co-culture as measured by LDH cytotoxicity assay when compared with autologous monocytes (*p* = 0.030 and *p* = 0.003, respectively; Figure 2a,b). There were varying degrees of target cell lysis, with undetectable levels of cytotoxicity in four of 12 co-cultures (Figure 2a). T cells, monocytes, or the CD45^―^ MPE fraction cultured alone produced minimal IFNγ (Figure 2b). IFNγ production varied, with five of 12 T cell co-cultures producing little to no IFNγ in the presence of autologous non-hematopoietic cell targets. Results from LDH release and IFNγ ELISA are reported in Table S1. CD137 (4-1BB) and CD134 (OX40) expression by CD8^+^ T cells following 24-h co-culture did not change significantly between the non-hematopoietic tumor containing co-cultures and monocyte controls (Figure S2). Results indicate that even with diverse disease and treatment status, a subset of patients possess MPE-derived CD8^+^ T cells which react to autologous tumor-containing target cells ex vivo.

### 2.3. Ex Vivo Expansion of MPE-Resident CD8^+^ T Cells Promotes an Exhausted Phenotype

**Figure 3:**
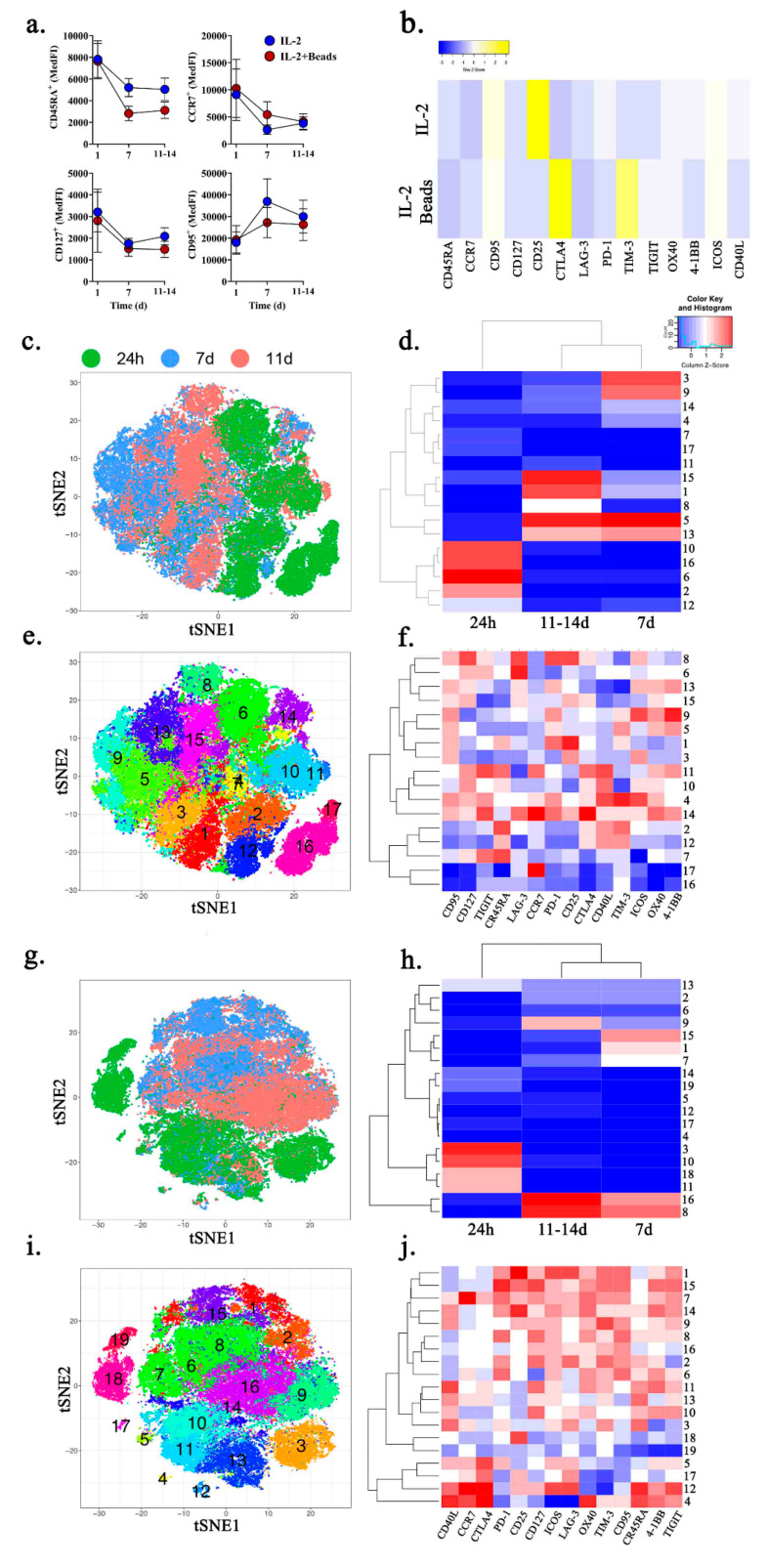
Ex vivo culture promotes terminal differentiation of CD8^+^ T cells from MPEs. CD8^+^ T cells were isolated via positive magnetic bead selection from six patients. T cells were cultured for 24 h, 7 days, or 11-14 days in either 6000 IU/IL-2 or 6000 IU/IL-2 and anti-CD3/CD28 activating microbeads, followed by cryopreservation, and simultaneous analysis by spectral cytometry. (**a**) Expression of individual markers of naïve cell state (CD45RA, CCR7, CD127) or maturation/exhaustion (CD95) in IL-2 (blue) or IL-2 and microbeads (red) treated cultures. (**b**) Change in expression levels of inhibitory and stimulatory co-receptors on T cells cultures in IL-2 or IL-2 and activating microbeads at day 7 compared to 24 h. t-SNE plots depicting combined data form 10,000 cells per patient per timepoint were generated for T cell cultures. Rphenograph clustering of unique subpopulations (**c,d**) and coding of T cells by culture timepoint at 24 h (green), 7d (blue), and 11-14d (red) (**e**,**i**) are shown for IL-2 only (top) and IL-2 with microbeads (bottom). Heatmaps depicting the distribution of Rphenograph clusters per culture timepoint are shown for (**g**) IL-2 and (**h**) IL-2 and microbead treated cells. Heatmaps of the corresponding factors used for description of the clusters (**f**,**j**).

To evaluate the effects of culture and rapid expansion of these MPE-resident CD8^+^ T cells (live CD45^+^CD3^+^CD4^−^CD8^+^), we employed multiparametric spectral flow cytometry to examine factors defining T cell memory (CD45RA, CCR7, CD127/IL-7Rα, CD25/IL-2R, CD95/Fas), inhibitory receptors (CD152/CTLA-4, CD223/LAG-3, CD279/PD-1, CD366/TIM-3, TIGIT), and co-stimulatory receptors (CD134/OX40, CD137/4-1BB, CD278/ICOS, CD154/CD40L). Bead-isolated MPE-resident CD8^+^ T cells from six patients were cultured in either 6000 IU/IL-2 or 6000 IU/IL-2 plus anti-CD3/CD28 activating microbeads (Dynabeads) at a 1:25 T cell to bead ratio for 24 h, 7 days, and 11-14 days. Anti-CD3/CD28 microbead activation resulted in substantial T cell expansion, serving as a surrogate for clinical rapid T cell expansion protocols (Figure S3). In both conditions there was a less differentiated phenotype at 24-h when compared to 7- and 11-14-day cultures. There was greater expression of CD45RA, CCR7, CD127, and lower expression of CD95 (Figure 3a). In the IL-2 only cultures, after 7 days there was significant upregulation of CD25, moderate upregulation of CD95, and decreases in all other examined factors (Figure 3b). By contrast, 7 days of culture in IL-2 plus anti-CD3/CD28 activating microbeads resulted in a significant increase in CTLA-4 and TIM-3 expression, moderate increase in CD95 expression, and downregulation of the remaining examined factors (Figure 3b).

Because the T cell compartment within MPEs is comprised of diverse memory subsets and functional states, we employed computational analysis for unbiased identification of unique subpopulations. Compiled T cell culture data from all patients and timepoints was used to generate t-SNE plots for data reduction and illustration. When analyzed with Rphenograph in Cytofkit [25], T cells at 24 h (green), 7 days (blue), or 11–14 days (red) separate into distinct groups irrespective of treatment conditions, demonstrating the impact of prolonged culture (Figure 3c,d,g,h). T cell cultures treated with IL-2 or IL-2 plus anti-CD3/CD28 activating microbeads segregated into 17 and 19 unique subpopulations, respectively (Figure 3e,f,i,j). In T cells cultured with IL-2 alone, there was enrichment of clusters 10, 16, 6, and 2 at 24 h (10, 16, 6, 2), but this changed at day 7 when it was clusters 3, 9, 5, and 13. At day 11–14, clusters 1 and 15 become prominent and clusters 5 and13 were conserved from day 7 (Figure 3d). Although each subpopulation is unique (Figure 3f,j), longitudinal culture results in progressive T cell alterations. There are early reductions in CD45RA, TIGIT, and LAG-3 but increased ICOS, OX40, and 4-1BB, followed by reductions in ICOS and 4-1BB. Similarly, IL-2 plus anti-CD3/CD28 activating microbeads results in the loss of clusters 3,10, 11 and 18 at 24 h but enrichment of clusters 8 and 16 after (Figure 3h). This progression is marked by early increased expression of CD95, TIM-3, and OX40 with decreased expression of CD45RA and CD40L, followed by loss of CD40L.

### 2.4. Acellular MPE Fluid Inhibits T Cell IFNγ Production and May Influence T Cell Phenotype

**Figure 4:**
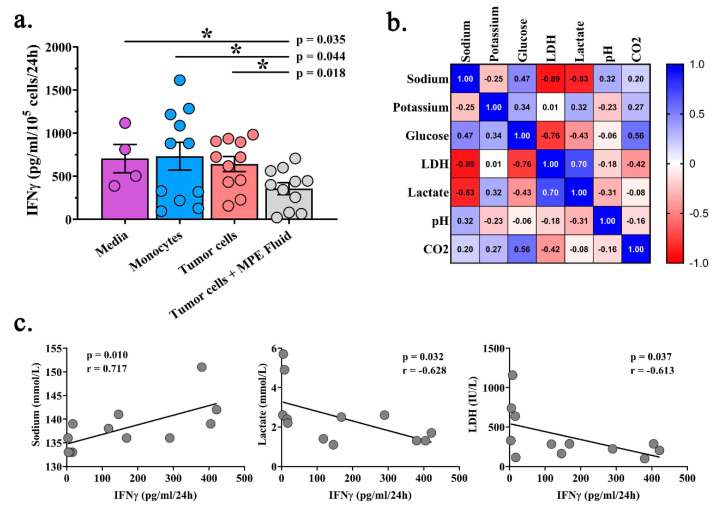
Acellular environment of MPEs is suppressive to CD8^+^ T cell function. CD8^+^ T cells were activated in the presence of anti-CD3/CD28 microbeads and IFNγ was measured in culture supernatants by specific ELISA following 24 h co-culture with media, autologous peripheral blood monocytes or autologous non-hematopoietic MPE cells. (**a**) IFNγ production by CD8^+^ T cells is significantly reduced upon culture in 50% autologous acellular MPE fluid compared to cells culture in media, with monocytes, or non-hematopoietic MPE cells. (**b**) Spearman correlations between levels of free analytes detected in acellular MPE fluid. (**c**) Spearman correlations between levels of sodium, lactate, and LDH identified in patient MPEs with patient matched CD8+ T cell production of IFNγ in response to co-culture with autologous non-hematopoietic MPE cells.

We next sought to examine the effect of the acellular component of MPE fluid on T cell function. MPE-resident CD8^+^ T cells were isolated and activated with anti-CD3/CD28 microbeads in the presence of IL-2, resulting in robust IFNγ production as detected by specific ELISA (Figure 4a). Co-culture of activated MPE-resident CD8^+^ T cells with autologous monocytes or tumor containing non-hematopoietic MPE cells did not affect IFNγ production. Notably, the anti-CD3/CD28 activated co-cultures plus 50% autologous acellular MPE fluid resulted in a significant reduction in IFNγ production (*p* = 0.018; Figure 4a). To investigate the acellular component of MPEs, cell-free MPE fluid was analyzed for sodium, potassium, glucose, LDH, lactate, pH, and CO_2_ (Table 3). There were increased levels of LDH, lactate, and glucose; reduced levels of CO_2_; and a basic pH when compared to normal reference values in serum. Levels of sodium within the MPE inversely correlated with both lactate and LDH (Figure 4b). We also examined the relationship between these factors and IFNγ production in these co-cultures. Interestingly, there was a positive correlation between CD8^+^ T cell production of IFNγ and sodium (*p* = 0.010) but an inverse correlation with lactate (*p* = 0.032) and LDH (*p* = 0.037; Figure 4c). These findings suggest that soluble components of the MPEs may suppress endogenous T cell function.

To further examine the relationship between the MPE environment and T cell phenotype, patients were grouped as high or low levels of sodium, lactate, and LDH (based on the median levels). Evaluation of subpopulations from the above t-SNE plots (Figure 3) identified distinct T cell phenotypes associated with both ex vivo culture conditions (Figure S4). These findings suggest that the MPE composition influences the dynamics and function of MPE resident CD8^+^ T cells.

### 2.5. Intra-Pleural MDSCs are Associated with Decreased Cytotoxic Function of MPE-Resident T Cells

**Figure 5:**
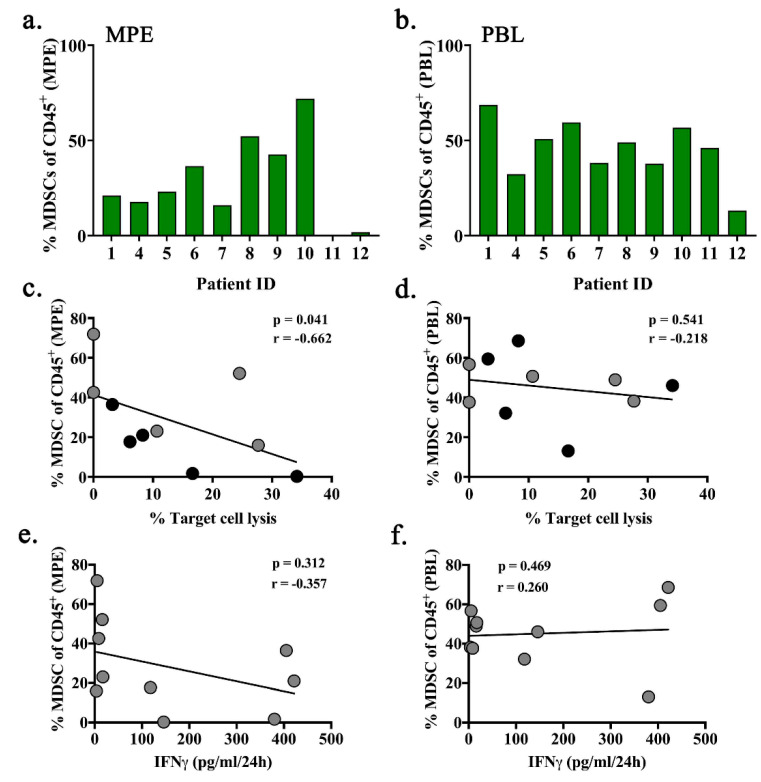
Intrapleural MDSCs are associated with reduced cytolytic capacity of MPE-resident CD8^+^ T cell. MDSCs were identified in MPE and PBL by flow cytometry as CD45^+^ SSC^High^ HLA-DR^Low^ CD14^+^ and/or CD15^+^ including both polymorphonuclear and monocytic subtypes. The proportion of MDSC shown as the percentage of total CD45^+^ leukocytes per patient are depicted for (**a**) MPE and (**b**) PBL. Spearman correlations between the percent of MDSCs in (**c**) MPE or (**d**) PBL and patient MPE-resident CD8^+^ T cell cytotoxicity upon co-culture with autologous non-hematopoietic MPE cells (highest value from co-cultures with either IL-2 alone [gray dots] or IL-2+anti-CD3/CD28 microbeads [black dots]). Spearman correlations between percent MDSCs in (**e**) MPE or (**f**) PBL and patient MPE-resident CD8+ T cell IFNγ production upon co-culture with autologous non-hematopoietic MPE cells.

Because immune composition may influence MPE-resident CD8^+^ T cell activity even after being removed from their proximity, we analyzed correlations with the previously described immune populations (Figure 1) as well as regulatory T cells (T_regs_), tumor-associated macrophages (TAMs), and myeloid derived suppressor cells (MDSCs). MPE-resident CD8^+^ T cells were co-cultured with autologous monocyte or tumor containing non-hematopoietic cells after 24-h incubation in IL-2 or IL-2 plus anti-CD3/CD28 activating microbeads. LDH cytotoxicity and IFNγ production were measured. The proportion of total MDSCs (including both M-MDSC (MHCII^Low^CD11b^+^CD66b^+^CD14^+^) and PMN-MDSC (MHCII^Low^CD11b^+^CD66b^+^CD15^+^)) in the MPEs was associated with decreased target cell lysis (*p* = 0.041; Figure 5a,c). By contrast, there was no correlation with the percentage of MDSCs in peripheral blood (*p* = 0.541; Figure 5b,d). There was also no correlation between percent MDSC in either MPE or peripheral blood and IFNγ production (Figure 5e,f). None of the remaining 11 leukocyte populations correlated with T cell function.

## 3. Discussion

Malignant pleural effusions have a high prevalence of tumor associated immune cells (up to 10^8^ T cells) that are readily and repeatedly accessible. While MPEs present a clinical problem, they also represent an opportunity to capture a broader and more reactive repertoire of T cells that are evolving against mutationally-driven neoepitope expressing tumor cells. In this study, the main findings are that eight of 12 MPEs from a heterogeneous population of cancer patients at different stages of treatment have T cells that can be activated 24 h after removal from the effusion, and this activity is possibly influenced by cellular and acellular components in the local compartment. Our finding that MPE-resident CD8^+^ T cells can mediate cytotoxicity toward the autologous non-CD45^+^ tumor containing fraction after only 24 h makes them a potential resource for finding a subset of tumor-specific T cells that are obtained in a minimally invasive manner. This could also be a useful screen for identifying patients that will respond to T cell targeted immunotherapies.

However, we do not know why some patients had minimal or no activation of their MPE-resident T cells in co-culture. One explanation is that not all MPEs contain tumor-specific CD8^+^ T cells, as can be seen with solid tumor. Measurements of anti-tumor reactivity vary depending on the cancer (melanoma, uveal melanoma, lung, bladder), the method for determining tumor infiltrating lymphocyte (TIL) reactivity (IFNγ production, CD137/4-1BB and CD134/OX40 expression), and the types of cells that are tested [26,27,28,29]. Because our sample size is small, future studies will allow us to better predict potential antitumor reactivity. Additionally, screening greater numbers of co-cultures, akin to multiple fragments of solid tumor, or pre-selecting specific populations may increase total anti-tumor reactivity [30,31]. Alternatively, because of variation in the tumor cell percentage that were present in the non-hematopoietic fraction, it is also possible that the stoichiometric presence of recognizable tumor antigens was too low to elicit significant T cell activation in some samples. Finally, there is heterogeneity in the types of cancers, systemic treatments, and clinical conditions that could impact T cell activity, which mimics clinical practice. Further investigation is needed to determine if there is a subset of MPE-resident T cells that are similar to traditional TIL.

Overcoming the immunosuppressive factors of the host is critical in potentiating immunotherapies. T_regs_, TAMs, and MDSCs are important contributors to the immunosuppressive tumor environment, and may play a role in pleural effusions [6,7,32,33]. Immunosuppressive cell populations were present within all 12 MPEs, but only the numbers of MDSCs correlated with the activation potential of CD8^+^ T cells in our series. Interestingly, MDSC-associated immunosuppression was observed after T cells were removed from their proximity in the MPE, suggesting that either MDSCs or factors associated with a MDSC-rich environment may impart lasting T cell dysfunction, perhaps through induced epigenetic effects. In addition to cellular suppression, an inhospitable environment plays a critical role in T cell biology that can lead to effector dysfunction [34]. Subsequently, we examined several molecules in the acellular fraction of MPEs to determine if there are derangements that may impact effector T cell activity. Our finding that sodium, LDH, and lactate levels correlate with activated CD8^+^ T cell IFNγ production is not surprising. Both LDH and lactate are markers of suppression and have been suggested to inhibit T cell migration and limit cytolytic function [9,35,36,37]. Additionally, sodium has been implicated in inhibiting the immunosuppressive function of T_regs_, however its role on CD8^+^ T cells is not well understood [38,39,40]. Though we found potential relationships with these three molecules, there is likely a complex interplay of many factors that contribute to the T cell dysfunction that we have not fully defined.

Because there is a low number of TILs obtained in solid tumors, these cells require expansion of roughly 800-fold to be utilized in ACT therapies, which can have several drawbacks [41,42,43]. Post-expansion TIL have been found to express markers suggestive of a more differentiated phenotype and an “activated state” after expansion [44,45]. A recent study examining TCRγ chain diversity in TIL from pancreatic ductal adenocarcinoma and melanoma demonstrated that ex vivo culture of TIL in rapid expansion protocols (REP) reduced diversity, and that expanded clonotypes present post-REP did not represent the same dominant clones identified within pre-expanded TIL cultures [46]. This work suggests that tumor antigen-specific TIL, likely the CD8^+^ PD-1^+^ subset, have decreased replicative fitness during ex vivo expansion due to their exhausted nature, and are thus out-competed by less differentiated clonotypes that lack tumor-specificity. The MPE-resident T cells that underwent culture and expansion for 7 or more days demonstrated progressive differentiation which may be associated with functional exhaustion. Our analysis confirmed distinct phenotypic populations were established over this period. However, we did not test if this has a consequence on T cell clonality, cytolytic activity, or IFNγ production. This phenomenon is suggestive of that seen in traditional TIL, however the ideal comparison would be to TIL from the same patients. There are obvious clinical barriers to obtaining metastatic solid tumor as well as MPE from the same patient, but is the subject of future studies. None the less, MPEs present a potential advantage of readily accessing “bulk” T cells with a less differentiated (pre-culture) state for studying tumor reactivity, or possibly ex vivo testing of T cell activating therapies [47].

Collectively, our findings beg the question if T cell repression in an MPE could be reversed. Future studies will explore effects of the immunosuppressive cells and acellular mediators on CD8^+^ T cells and may inform us of the short-and long-term alterations they influence. Additionally, because the pleural cavity can be controlled (to some degree) with an indwelling catheter, perhaps it indicates a need to drain or modify the composition in order to potentiate immunotherapies so that new tumor reactive T cells can be recruited to a more hospitable environment.

There are several limitations to this study. First, this study was of a small population of patients on a variety of chemo and immune therapies with heterogeneous cancers. All of these may alter the composition of the effusion, including the cells and soluble factors. This may have also altered the response of the CD8^+^ T cells in the cytotoxicity assays. At the same time, this complex and diverse population is an accurate representation of patients seen in clinical practice who may benefit from advanced immunotherapy. Additionally, we were unable to directly compare the MPE resident T cells to traditional TIL, as the MPE patients were undergoing palliative procedures and solid tumor from these same patients was not available. Future experiments will involve comparisons to TIL and PBMC from either patients undergoing ACT with traditional TIL or other solid tumor resections. CD137/4-1BB and CD134/OX40 expression was not significantly elevated after 24-h of co-culture, bringing into question if the cytotoxicity is MHC restricted. This will be the topic of future studies as unrestricted αβ and γδ T cell and NK cell killing could account for the observed cytotoxicity.

Strategies that activate tumor-reactive T cells for the treatment of patients with advanced solid cancers have transformed the therapeutic landscape. We are only beginning to understand the complex interactions that take place within the tumor environment that may promote tumor growth and ultimately prohibit the control of disease. MPEs are an underutilized resource that is usually discarded, but instead could be studied to understand immunosuppressive factors that can be modified for better effector T cell function with immunotherapy.

## 4. Materials and Methods

### 4.1. Collection of Specimens

Informed consent for patient participation in this study was obtained prior to drainage from all patients, and no subjects were under the age of 18. The use of human tissue samples in the experiments described were approved by the institutional review board at the University of Pittsburgh (IRB#PRO16110093). Samples were collected as excess pathologic specimens and experiments were not performed on humans. All methods were carried out in accordance with relevant guidelines and regulations.

A total of 12 MPEs (300 cc–1000 cc) were collected for clinically indicated drainage of symptomatic effusions, either by thoracentesis, or from a temporary or indwelling tunneled catheter. These specimens would otherwise be medical waste. Cell counts were performed prior to sample processing. MPEs were centrifuged to pellet cells, and acelluar fluid was collected and stored at −80 °C. The cellular fraction was then lysed of red blood cells, washed, counted, sorted, and utilized for co-culture assays and phenotypic analysis. All effusions were examined by a cytopathologist to confirm the presence of malignant cells.

### 4.2. T Cell Expansion and Co-Cultures

The cellular component of MPEs were collected and red blood cells removed by hypotonic lysis. CD45^―^ non-hematopoietic tumor containing cells and CD45^+^CD8^+^ T cells were isolated by negative and positive magnetic bead enrichment, respectively (BioLegend, San Diego, CA, USA). CD14^+^ monocytes were isolated from patient matched peripheral blood by positive bead enrichment (BioLegend, San Diego, CA, USA). T cells were cultured for up to 14 days in complete AIM-V media containing 5% human serum supplemented with 6000 IU/mL IL-2 (StemCell Technologies, Vancouver, Canada) or 6000 IU/mL IL-2 with anti-CD3/CD28 T cell activating beads (Dynabeads, LifeTechnologies, Carlsbad, CA, USA; 1:25 microbead to T cell ratio). Anti-CD3/CD28 beads were used to mimic conditions in an expansion protocol for adoptive cell transfer therapy. Media was exchanged every 2–3 days to replenish IL-2. For co-culture assays, the tumor containing portion was the CD45^―^ fraction, which contained tumor cells, mesothelial cells, and a small percent of “other” cells. The CD8^+^ T cells were derived from the CD45^+^ fraction with bead enrichment. MPE-derived T cells were cultured as above for 24 h prior to the addition of either autologous non-hematopoietic tumor containing cells or autologous peripheral blood monocytes. Co-cultures containing a 1:1 ratio of 10^5^ T cells with tumor containing cells or monocytes in 96-well flat-bottomed plate were incubated for 24 h. Cell-free supernatant was collected and remaining cells harvested for flow cytometry analysis. Because of limited cells, seven of 84 co-culture conditions were not performed (4 monocyte and 3 tumor co-cultures). For T cell expansion, cells were cultured and/or expanded for 24 h, 7 days, or 11–14 days then cryopreserved for analysis.

### 4.3. LDH Cytotoxicity Assay

Cytotoxicity was measured in cell-free co-culture supernatant using a lactose dehydrogenase (LDH) cytotoxicity assay kit (Pierce, Thermo Scientific, Waltham, MA, USA) per manufacturer’s instructions. Maximum LDH release was determined by treatment of cells (CD45^―^ CD8^―^ MPE cells or monocytes) with manufacturer’s lysis buffer. Spontaneous LDH release measured from T cells, monocytes, and CD45^―^CD8^―^ MPE cells cultured alone was subtracted from corresponding co-culture values. Percent cytotoxicity was calculated as the experimental value minus the T cell spontaneous release value minus the target cell spontaneous release value divided by the target cell maximum release post-lysis value minus target cell spontaneous release control times 100. Assays were performed in duplicate.

### 4.4. IFNγ Elisa

Interferon gamma (IFNγ) release was measured in 50 µL of cell-free co-culture supernatant following T cell co-cultures using an IFNγ-specific ELISA antibody pair and buffer kit (Invitrogen) per manufacturer’s instructions. Assays were performed in duplicate with manufacturer provided IFNγ standard controls.

### 4.5. Flow Cytometry

Immunophenotyping of MPE and matched peripheral blood were performed on freshly isolated samples. Phenotypic characterization of ex vivo expanded CD8^+^ T cells was performed at the same time following cryopreservation to eliminate batch effects. All reagents were purchased from BioLegend unless specified. 1–5 × 10^6^ cells per sample were stained in Cell Staining Buffer using combinations of mAbs specific followed by labeling with amine-reactive viability dye (LiveDead, Molecular Probes, Eugene, OR, USA). To determine leukocyte composition in MPE and blood, cells were labeled with mAbs for: EpCAM (9C4), CD45 (HI30; BD Biosciences, San Jose, CA, USA), CD3 (UCHT1), CD4 (RPA-T4; Invitrogen, Carlsbad, CA, USA), CD8a (RPA-R8), HLA-DR (L243), CD11b (ICRF-44), CD14 (HCD-14), CD16 (3G8), CD15 (W6D3), CD66b (G10F5; BD Biosciences), CD123 (6H6), CD11c (3.9), CD56 (HCD56; BD Biosciences), CD19 (SJ25C1), and TCR γ/δ (B1). Expanded T cell cultures were analyzed for: CD3 (UCHT1; BD Biosciences), CD4 (RPA-T4), CD8a (RPA-T8), CD45RA (HI100), CD197 (G043H7), CD95 (DX2), CD25 (BC96), CD127 (A019D5), CD152 (BNI3; BD Biosciences), CD223 (Bristol-Myers Squibb, New York, NY, USA), CD279 (eBioJ105; eBioscience, San Diego, CA, USA), CD366 (7D3; BD Biosciences), TIGIT (A15153G), CD134 (Ber-ACT35), CD137 (4B4-1; BD Biosciences), CD278 (ISA-3; eBioscience), CD154 (24-31), and CD357 (108-17). Samples were fixed in 1% paraformaldehyde and data were collected on a five laser LSRFortessa (BD Bioscience) or four laser Cytek Aurora spectral cytometer. FlowJo (BD) software was used for conventionally gated data analysis. Lineage for DC identification consisted of CD3^+^, CD19^+^, and CD56^+^ cells. For computational analysis, samples were analyzed using Cytofkit package for R studio as previously described [25]. Briefly, CD8^+^ T cells were manually gated as live, single, CD3^+^ CD4^−^ CD8^+^ cells using FlowJo. The gating strategy used to measure lymphoid and myeloid cell subsets is shown in Figure S5. Shown is the successive gating strategy used to ultimately visualize individual cell types from sized, single, viable CD45^+^ leukocytes. Specific data from flow cytometry experiments are available upon request. Preprocessing was performed to generate expression matrix for each sample in a Flow Cytometry Standard (FCS) file. Parameters of interest were selected and FSC files were exported and uploaded into Cytofkit package. FCS files were transformed using automatic logicle transformation (autoLgcl) and merged in to one matrix using ceil. Cells were clustered using Rphenograph and visualized using t-Distributed Stochastic Neighbor Embedding (t-SNE). This is a graph-based partitioning method which dissects nearest-neighbor data into phenotypically coherent populations based on relatedness. Samples were grouped by time points 24 h, 7 days and 11–14 days and displayed using t-SNE. Heatmaps were generated from Rphenograph using the expression heat map option depicting Rphenograph clusters and marker expression per cluster.

### 4.6. Clinical Chemistries

Clinical chemistries (sodium, potassium, carbon dioxide, glucose, pH, lactate, and lactate dehydrogenase) were obtained from frozen samples of the acellular fraction of malignant pleural effusions through the clinical laboratories at the University of Pittsburgh Medical Center per their protocols.

### 4.7. Statistical Analysis and Scientific Rigor

All results were expressed as means ± standard error of the mean (SEM) unless otherwise stated. Data were analyzed using non-parametric Mann-Whitney U tests for comparisons of patient groups, unpaired Student’s t-test for analysis of T cell culture experiments, or Spearman rank-order correlation tests performed using GraphPad Prism8 (GraphPad Software, San Diego, CA, USA). For all hypothesis tests, a *p* < 0.05 was considered statistically significant.

## Figures and Tables

**Table 1:** Tumor types and clinical characteristics from 12 malignant pleural effusions.

Patient	Tumor Origin	Patient Age (years)	Total Volume Collected (L)/Cellularity (cells/L)	Treatment at Drainage	Prior Treatments	Survival from Initial Drainage (days)
1	Ovarian	68	1.0/8.00 × 10^8^	olaparib	doxorubicin, taxol	231
2	Pancreatic *	70	0.7/5.63 × 10^8^	TRX518/gemcitabine	FOLFIRINOX; gemcitabine/nab-paclitaxel	42
3	Pancreatic *	70	0.6/5.29 × 10^8^	TRX518/gemcitabine	FOLFIRINOX; gemcitabine/nab-paclitaxel	42
4	Lung	62	0.5/2.32 × 10^8^	dabrafenib/trametinib	none	198
5	Breast	62	0.6/8.68 × 10^8^	nab-paclitaxel/herceptin	pertuzumab; trastuzumab; paclitaxel; anastrozole; letrozole; tamoxifen; exemestane	185
6	Lung (SCLC)	68	0.5/1.36 × 10^9^	ipilimumab/nivolumab	AEB1102; rovalpituzumab teserine; nivolumab; carboplatin/etoposide; topotecan	45
7	Salivary	74	0.3/6.67 × 10^10^	cisplatin/cyclophosphomide/doxorubicin	carboplatin/taxol; lupron; pembrolizumab	7
8	Gastric	49	0.7/2.32 × 10^8^	paclitaxel/ramucirumab	FOLFOX; XELIRI and pembrolizumab	13
9	Lung (SCC)	77	0.5/1.85 × 10^8^	pembrolizumab/nab-paclitaxel	none	10
10	Lung (AC)	46	1.0/1.02 × 10^8^	alcetinib	none	117
11	Lung (NEC)	61	0.4/2.2 × 10^9^	carboplatin/etoposide/atezolizumab	lanreotide; carboplatin/etoposide	107
12	Lung (AC)	91	0.7/2.03 × 10^8^	none	none	8

SCLC—small cell lung cancer; SCC—Squamous cell cancer; AC—adenocarcinoma; NEC—neuroendocrine carcinoma; L—liters. * Represents a single patient with bilateral malignant pleural effusions.

**Table 2:** MPE/PBL Correlations of Immune Composition.

Population	MPE (mean±SEM)	PBL (mean±SEM)	Spearman r	*p*-Value
Neutrophils	29.24 ± 9.891	68.66 ± 6.462	0.5758	0.0883
Macrophages	8.11 ± 1.874	5.97 ± 2.232	0.4667	0.1786
Monocytes	15.00 ± 6.267	8.33 ± 3.092	0.8545	0.0029
NK cells	2.77 ± 1.336	1.06 ± 0.503	0.1520	0.6738
B cells	6.66 ± 1.947	4.82 ± 3.141	0.7576	0.0149
pDCs	0.209 ± 0.176	0.024 ± 0.012	−0.2330	0.5240
mDCs	0.044 ± 0.013	0.071 ± 0.031	−0.6123	0.0649
CD8^+^ T cells	11.09 ± 1.600	5.598 ± 1.425	0.1879	0.6073
CD4^+^ T cells	26.87 ± 7.413	5.445 ± 1.654	0.4061	0.2475

**Table 3:** Chemical analysis of acellular MPE fluid.

Patient	Tumor Origin	Sodium (mEq/L)	Potassium (mEq/L)	Glucose (mg/dL)	LDH (IU/L)	Lactate (mEq/L)	pH	CO_2_ (mEq/L)
1	Ovarian	142	3.3	101	202	1.7	8.3	20
2	Pancreatic *	136	4.3	126	286	2.5	7.9	26
3	Pancreatic *	136	4.3	125	221	2.6	8.0	26
4	Lung	138	4.1	106	282	1.4	8.1	25
5	Breast	139	4.5	164	113	2.2	8.1	26
6	Lung (SCLC)	139	3.9	101	286	1.3	8.1	22
7	Salivary	136	3.8	80	328	2.6	8.0	33
8	Gastric	133	3.7	102	636	2.4	8.2	24
9	Lung (SCC)	133	4.4	55	1158	4.9	8.3	24
10	Lung (AC)	133	4.0	85	739	5.7	7.9	21
11	Lung (NEC)	141	3.9	105	161	1.1	8.0	25
12	Lung (AC)	151	3.9	134	98	1.3	8.3	38
	MPE Values (mean ± SEM	138.1 ± 1.5	4.0 ± 0.1	107 ± 8.1	375.8 ± 90.7	2.5 ± 0.42	8.1 ± 0.04	25.8 ± 1.5
	Normal serum reference	136–146	3.5–5.0	70–99	<171	0.5–1.6	7.35–7.45	35–45

* Represents a single patient with bilateral malignant pleural effusions.

## Supplementary Materials

**Supplementary Materials** can be found at https://www.mdpi.com/1422-0067/21/17/6178/s1. Figure S1: Percent of effector cells isolated from the CD45^-^ fractions of the MPE; Figure S2: CD8+ T cell expression of CD137/4-1BB and CD134/OX40 following 24-h of co-culture with the non-hematopoietic tumor containing fraction from MPEs; Figure S3: T cell expansion that occurred after culture with CD3/CD28 microbead activation; Figure S4: T cell phenotypes that resulted after ex vivo culture with and without CD3/CD28 microbeads for 24 h, 7 days, or 11-14 days; Figure S5: Gating strategy for to measure lymphoid and myeloid cell subsets during flow cytometry analysis; Table S1: Functional Activity of 24-h co-cultured CD8^+^ T cells and autologous CD45^−^ tumor containing fraction.

## Abbreviations

MPE	Malignant pleural effusion
ACT	Adoptive cell transfer
TAM	Tumor associated macrophage
LDH	Lactose dehydrogenase
IFNγ	Interferon gamma
FCS	Flow cytometry standard
t-SNE	t-distributed stochastic neighbor embedding
SEM	Standard error of the mean
Tregs	Regulatory T cells
TIL	Tumor infiltrating lymphocytes
