# Supplementary material for: Characteristics of Malignant Pleural Effusion Resident CD8+ T Cells from a Heterogeneous Collection of Tumors
Source: Int J Mol Sci. 2020 Aug 27;21(17):6178. doi: 10.3390/ijms21176178 (PMC7503595; doi:10.3390/ijms21176178)
Supplement: Supplementary file 1 [file ijms-21-06178-s001.pdf]

Figure S1: Percent of effector cells isolated from the CD45⁻ fractions of the MPE.

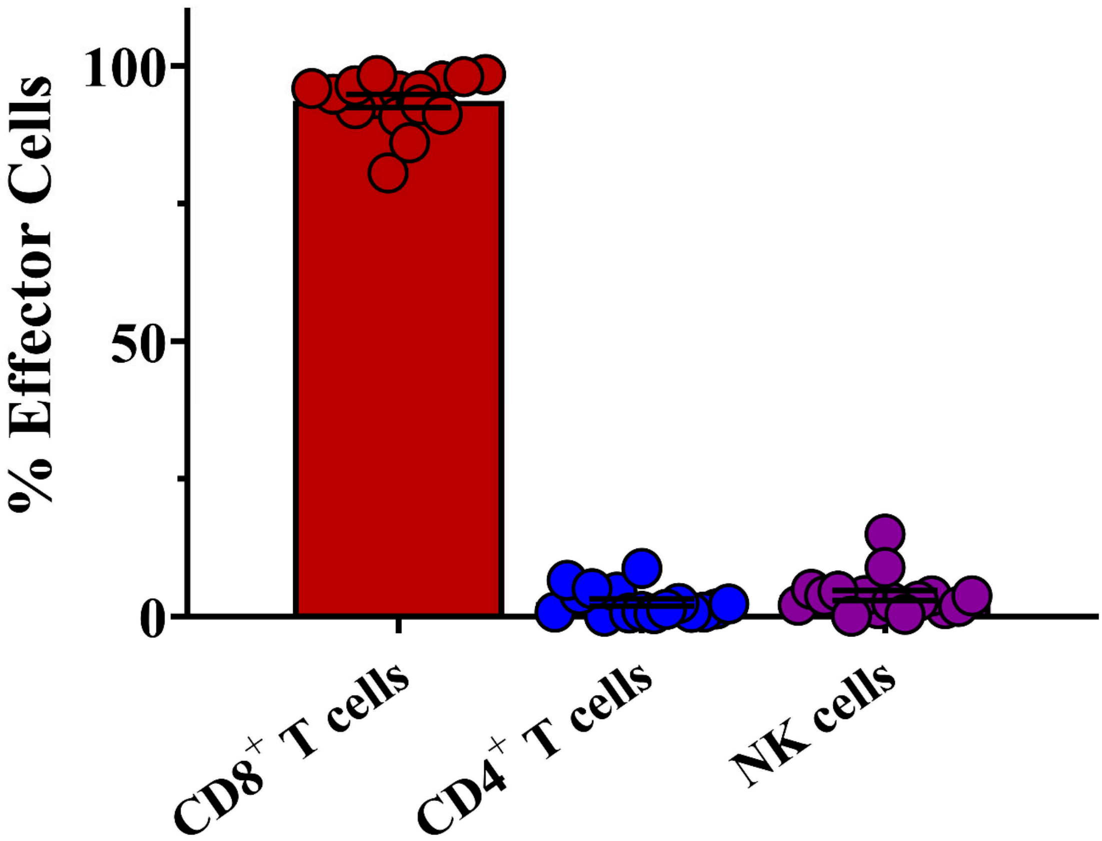

Figure S2: CD8+ T cell expression of CD137/4-1BB and CD134/OX40 following 24-hours of co-culture with the non-hematopoietic tumor containing fraction from MPEs.

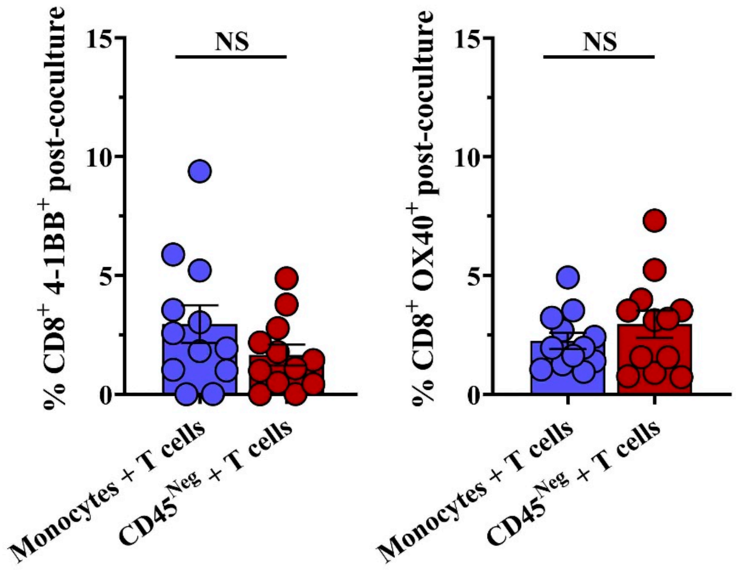

Figure S3: T cell expansion that occurred after culture with CD3/CD28 microbead activation.

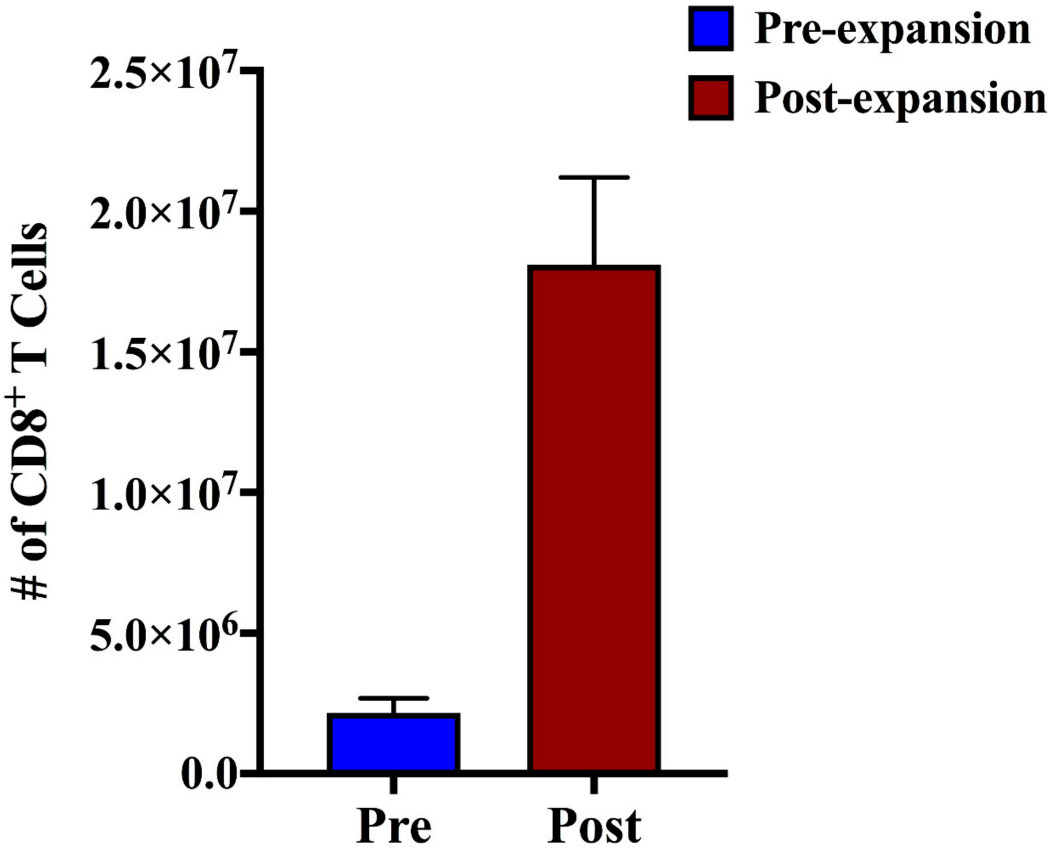

Figure S4: T cell phenotypes that resulted after ex vivo culture with and without CD3/CD28 microbeads for 24 hours, 7 days, or 11-14 days. Conditions were either high or low (based on median) levels of sodium, lactate, and LDH.

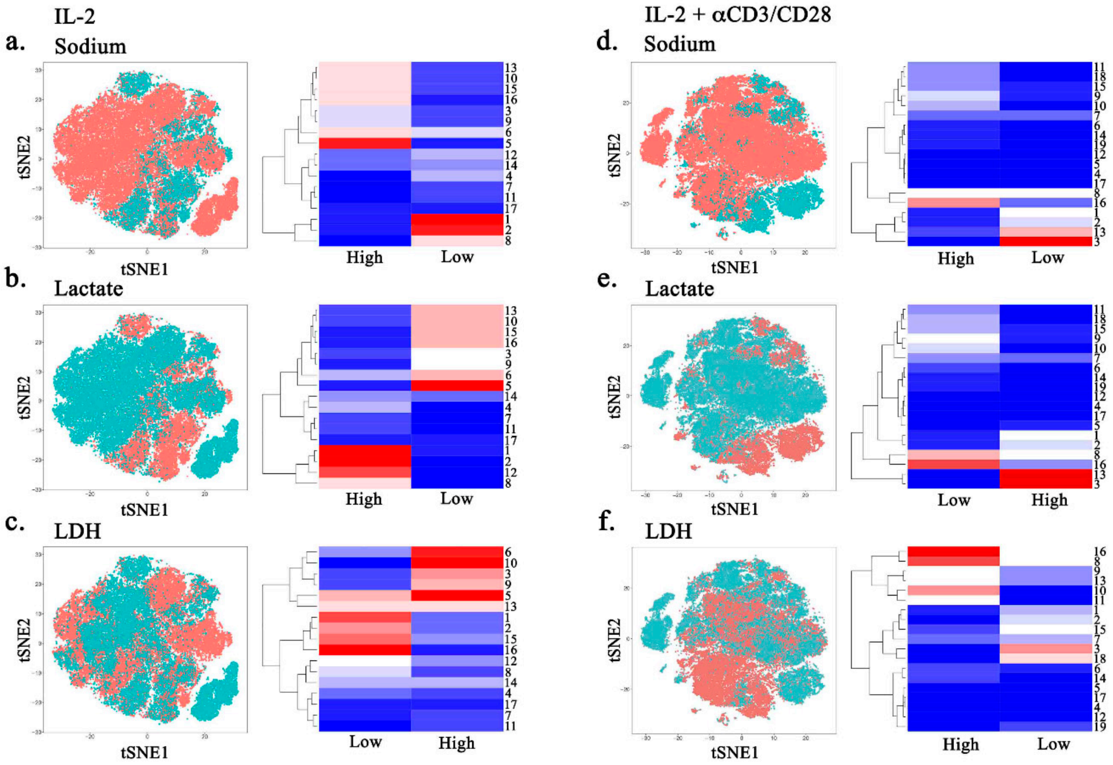

Figure S5: Gating strategy for to measure lymphoid and myeloid cell subsets during flow cytometry analysis.

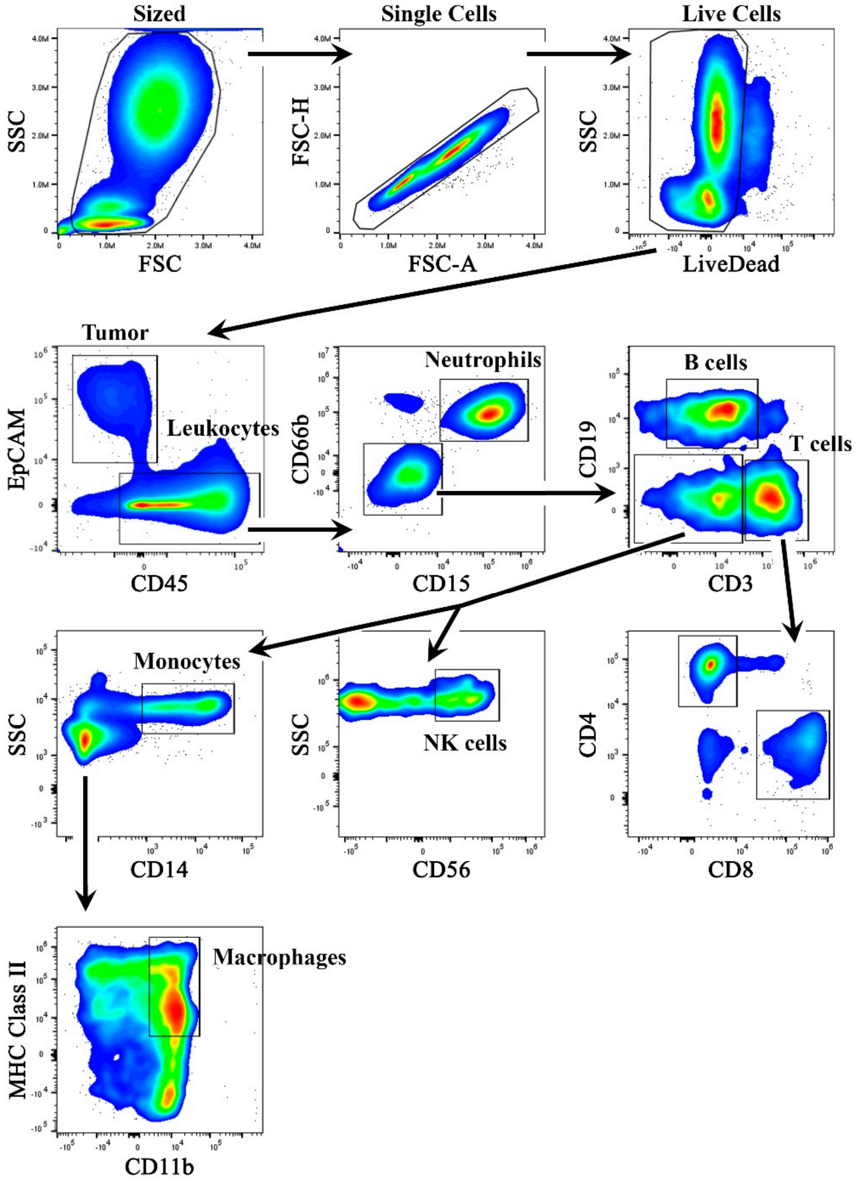

Supplementary Table 1: Functional Activity of 24-hour co-cultured CD8^{+} T cells and autologous CD45^{-} tumor containing fraction. LDH release in co-culture (IL-2 only, or IL-2 + anti-CD3/CD28 microbeads), interferon gamma production by T cells in co-culture (IL-2 only or IL-2 + anti-CD3/CD28 microbeads), and percent tumor cells in the CD45^{-} component of the MPE.

Patient	LDH release[a] (%)	LDH release[b] (%)	IFNγ [a] (pg/ml)	IFNγ [b] (pg/ml)	CD45^{Neg} (%)[c]	Tumor (%) of CD45^{Neg}
1	3	8	422	896	90	33.3
2	0	17	169	935	90	70.0
3	0	0	290	433	90	60.0
4	5	6	83	723	75	20.0
5	11	0	17	227	25	50.0
6	3	3	381	721	35	33.3
7	8	0	4	454	95	94.4
8	25	5	16	156	95	94.1
9*	0	0	8	983	NA	NA
10	0	NA	5	NA	15	100
11	16	34	116	905	45	50.0
12	12	17	380	617	60	33.3

IFNγ – interferon gamma; NA – not available

[a] IL-2 only

[b] IL-2 + anti-CD3/CD28 microbeads

[c] total percent CD45 negative cells in MPE (tumor + mesothelial cells + fibroblasts)

* percent tumor not able to be verified as pathology was determined at an outside institution
